# Acute Airway Crisis in Mucopolysaccharidosis VI: Management Challenges

**DOI:** 10.3390/diagnostics16071009

**Published:** 2026-03-27

**Authors:** Assel Tulebayeva, Chaitanya Gadepalli, Maira Sharipova

**Affiliations:** 1 Paediatric Department, Asfendiyarov Kazakh National Medical University, Almaty 050012, Kazakhstan; asselatulebayeva@gmail.com; 2 Scientific Center of the Pediatrics and Pediatric Surgery JSC, Almaty 050060, Kazakhstan; 3 Salford Care Organisation, Northern Care Alliance NHS Foundation Trust, The University of Manchester, Manchester M6 8HD, UK; cgadepalli@gmail.com

**Keywords:** mucopolysaccharidosis VI, airway management, crisis

## Abstract

**Background and Clinical Significance:** Mucopolysaccharidosis type VI is a rare lysosomal storage disorder due to arylsulfatase B enzyme deficiency, leading to progressive multisystem disease and complex airway. Acute respiratory infections can precipitate airway embarrassment. A structured treatment guideline is currently lacking. We present a 7-year-old MPS VI male with respiratory distress, highlighting the challenges in management. **Case Presentation:** Case review focusing on clinical presentation, imaging findings, and multidisciplinary decision-making during acute deterioration. A child diagnosed with MPS VI at the age of 3.5 years old, due to low arylsulfatase B enzyme activity and homozygous for c.275C>A p.(Thr92Lys) variant in the *ARSB* gene. At 7 years of age, he showed the signs of dyspnoea, increased respiratory effort with bilateral crepitations, and noisy breathing. Initial management included facemask oxygen, nebulised adrenaline, corticosteroids, and bronchodilators. Computer tomography scan of the neck and chest showed a complex upper airway, multiple tracheal narrowing, tortuosity, and an extra loop of truncus brachiocephalicus from the arch of the aorta. Potential interventions carried substantial risks due to abnormal airway and multisystem disease. Following extensive multidisciplinary discussion after careful consideration of the significant risks associated with invasive airway interventions, a shared decision was reached with the family to adopt a comfort-focused palliative care approach. Despite the best supportive care, the child unfortunately passed away after 3 months. The family was involved in every decision process and was fully supported. **Conclusions:** MPS VI is associated with complex airways and multisystem disease. Multidisciplinary decision-making with family is critical to safe and appropriate care. The rarity of the disease, lack of guidelines, complex airways, and multiple comorbidities make management challenging.

## 1. Introduction and Clinical Significance

Mucopolysaccharidoses (MPS) are rare, inherited, lysosomal storage diseases with a combined incidence of 1 in 22,000 [1]. They arise due to a deficiency of lysosomal hydrolases, leading to the accumulation of glycosaminoglycans (GAGs) in various parts of the body. Depending on the type of enzyme deficiency, MPS can be classified into 8 different types [2]. Table 1 shows various types of MPS, with enzyme deficiency, incidence, inheritance and life expectancy [3].

MPS type VI or Maroteaux-Lamy syndrome is one of the MPS; it is a rare autosomal recessive inherited lysosomal disorder with a birth prevalence of between 1 in 43,261 and 1 in 1,505,160 live births [5]. According to epidemiological data, Maroteaux-Lamy syndrome is the third most common MPS type in Kazakhstan, with a birth prevalence of 0.12 per 100,000 live births [8]. The disease develops due to a defect in the Arylsulfatase B (*ARSB*) gene coding for the enzyme lysosomal hydrolase N-acetylgalactosamine 4-sulphatase, leading to the accumulation of GAGs such as dermatan sulphate and chondroitin sulphate in various parts of the body, causing multiorgan damage. MPS VI affects multiple systems, including cardio-vascular, respiratory and skeletal systems, but neuro-cognitive aspects are normally preserved in this type of MPS [9,10]. Respiratory system impairment is one of the leading causes of early mortality in MPS. Patients with MPS VI are known to have complex airways [9,11]. Abnormalities in the airway and ventilation can arise from the lips to the lungs. In the upper airways, these patients may have bulky upper airways and limited neck extension; mouth opening can make access to the airways difficult. Angulation in the trachea, stenosis, malacia and tortuosity can make navigation through the airway difficult. Often, these patients also have reduced lung capacity, pulmonary changes, and splinting of the diaphragm due to hepatosplenomegaly, making ventilation challenging. Musculoskeletal problems in the cervical, thoracolumbar spine, and rib cage can add to the existing airway issues. Often these patients also have sleep disordered breathing, obstructive sleep apnoea [9]. Respiratory tract infections in this scenario may become challenging in an already compromised airway. We discuss an MPS VI diagnosed at the age of 3 months, now 7 years of age, presenting with airway embarrassment following a respiratory tract infection. We discuss the various management methods, dilemmas and ethical issues in the management of this complex situation by various health care professionals.

A detailed case review was undertaken, examining the child’s clinical presentation, progression of respiratory symptoms, and bedside assessment during acute deterioration. Diagnostic evaluation included analysis of cross-sectional imaging and three-dimensional airway reconstructions to characterise structural abnormalities contributing to respiratory compromise. The review also explored the multidisciplinary discussions involving respiratory, ENT (Ear, nose and Throat), anaesthetic, and palliative care teams, focusing on risk–benefit considerations and shared decision-making with the family.

## 2. Ethical Issues

This case report was prepared in accordance with ethical standards for clinical case reporting. Written informed consent for publication was obtained from the patient’s family, including consent to publish clinical details and imaging findings. All identifying information has been removed to ensure full confidentiality. As this is a single retrospective case report with no experimental intervention, formal ethical approval was not required according to local regulations. The authors affirm that the report adheres to the principles of the Declaration of Helsinki.

## 3. Case Presentation

The patient was diagnosed with mucopolysaccharidosis type VI (MPS VI) at 3 years of age following identification of markedly reduced arylsulfatase B activity (2.7 μmol/L/h; normal ≥8.8 μmol/L/h). Genetic testing confirmed a homozygous pathogenic *ARSB* variant, c.275C>A p.(Thr92Lys). According to the Human Gene Mutation Database (HGMD) Professional 2018.1, this variant has previously been described as disease-causing for MPS VI by Karageorgos et al., 2007 [12]. At diagnosis, he exhibited early phenotypic features including coarse facial characteristics, short stature (height z-score −2.37), dysostosis multiplex, pectus carinatum, generalised hypotonia, and multiple Mongolian spots. Additional findings included mild corneal clouding, hypertrophied adenoids and tonsils, and an asymptomatic umbilical hernia. Hearing and dentition were normal. Parents reported nasal obstruction and snoring, although there were no witnessed apnoeic episodes or daytime somnolence. Overnight oxygen saturations occasionally fell below 96%, but formal polysomnography could not be performed. Enzyme replacement therapy (ERT) was initiated promptly, and he underwent regular multisystem surveillance over the following four years. His comorbidities included asthma, and he had documented allergies to co-enzyme Q and herbal preparations used for sinusitis. At 7 years of age, he was admitted with increasing shortness of breath, recurrent respiratory infections, and worsening sleep quality associated with snoring. His exercise tolerance had declined; he remained independently mobile indoors but required assistance outdoors. There were no acute ENT, ophthalmic, cervical spine, or cognitive concerns.

On examination, he remained markedly short for age (height 99 cm, z-score −4.76), with weight 19 kg (z-score −1.85) and BMI 19.4 (z-score 1.62). He was afebrile. Oxygen saturations on room air fluctuated significantly, with a nadir of 50%. Although prior admission, there was no clear history of obstructive sleep apnoea, the combination of snoring and nocturnal desaturations suggested possible undiagnosed sleep-disordered breathing. Respiratory examination revealed tachypnoea (24 breaths/min), increased work of breathing with accessory muscle use, bilateral chest crepitations, and noisy breathing. Pulmonary function testing demonstrated severe restrictive impairment reflected in the Forced Vital Capacity (FVC—20%) and Forced Expiratory Volume in one second (FEV1—23%). Cardiac examination showed relative cardiac dullness extending 0.5 cm beyond the left midclavicular line, muffled heart sounds, a systolic murmur at the apex, and tachycardia (125 beats per minute). Echocardiography revealed mitral valve cusp thickening with moderate regurgitation, left ventricular hypertrophy, an aortic fibrous ring, and preserved ejection fraction (65%). Haematological (full blood count and C-reactive protein) and coagulation parameters were normal.

**Figure 1:**
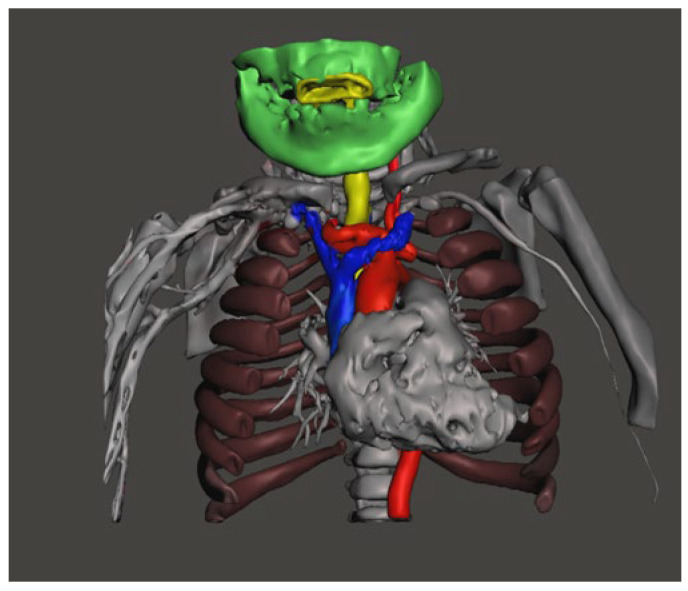
Three-dimensional reconstruction anteroposterior view of the lower head, chest showing disproportionately large skull (green) in relation to chest (brown), short neck, broad ribs (brown), large heart (grey) and reduced space for lungs. The trachea is shown in yellow, ascending aorta and large arteries in red, major veins in blue.

**Figure 2:**
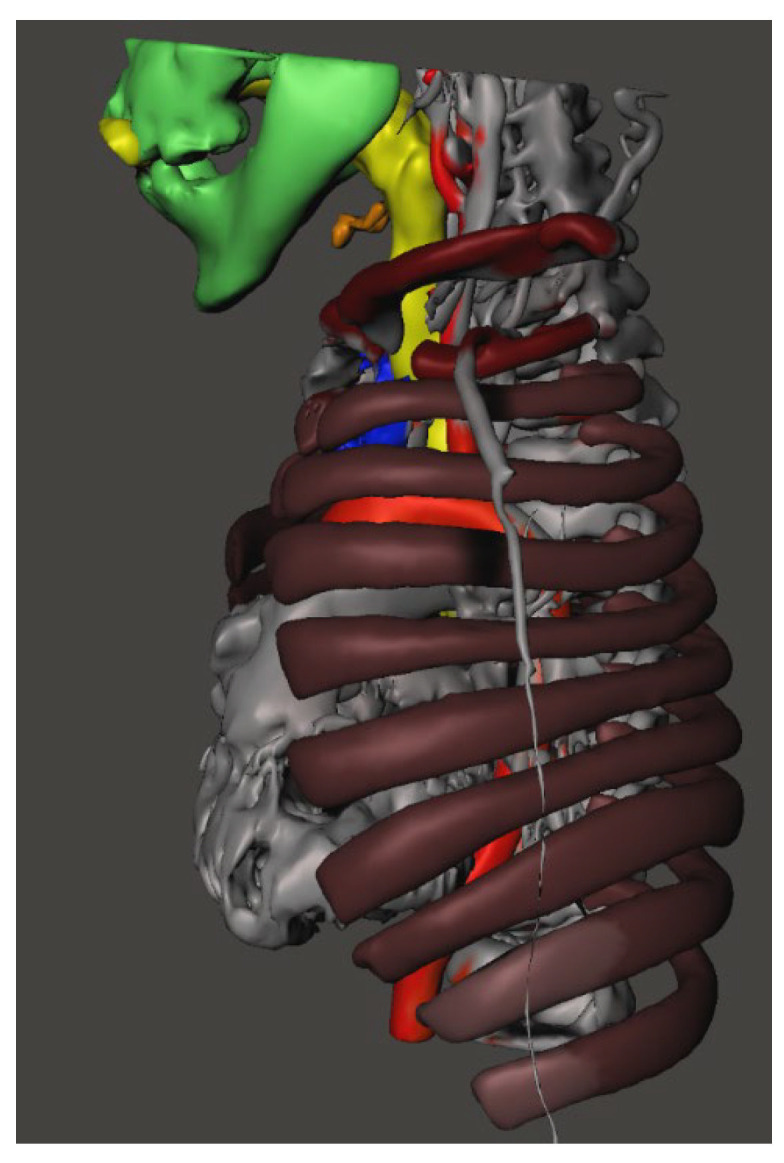
3-dimensional reconstruction of the lower head, chest showing short neck, large jaw, hyoid bone tucked under the jaw, broad spatulate ribs and reduced intercostal spaces.

**Figure 3:**
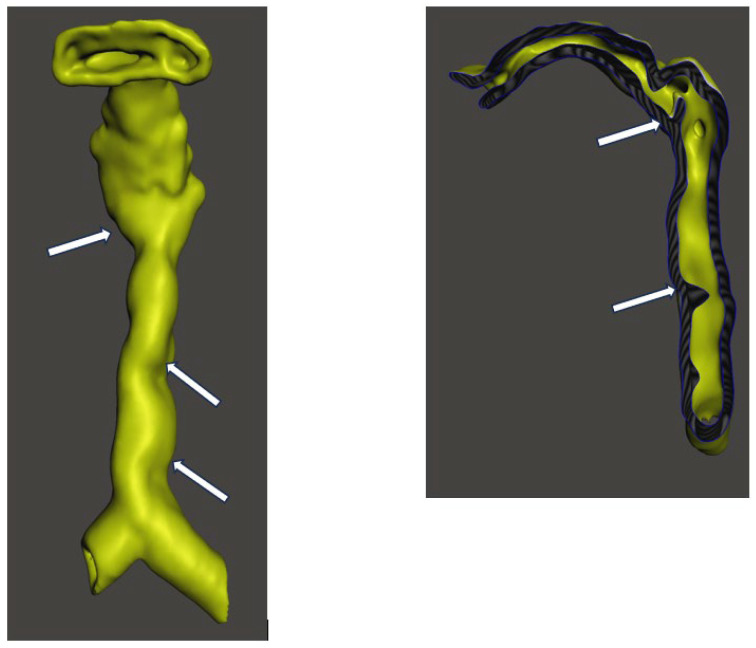
3D reconstruction of anteroposterior and left lateral cross-section view of the oropharynx, larynx and trachea showing high larynx where the epiglottis is marked with an arrow, multiple areas of tracheal narrowing marked with arrows.

**Figure 4:**
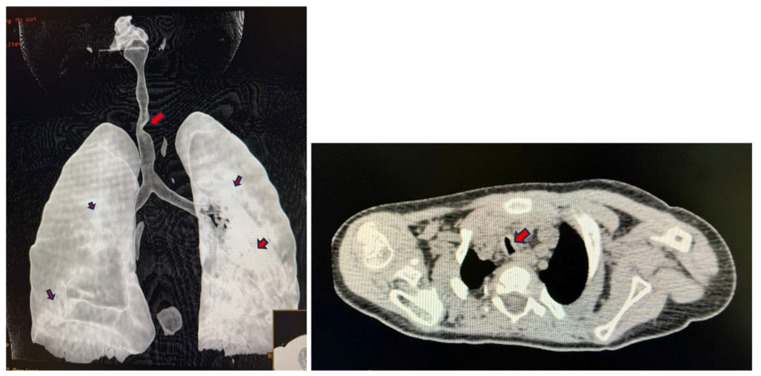
CT scan of chest and 3-dimensional reconstruction shows tortuous and narrowed trachea and patchy infiltrates marked by red arrows.

**Figure 5:**
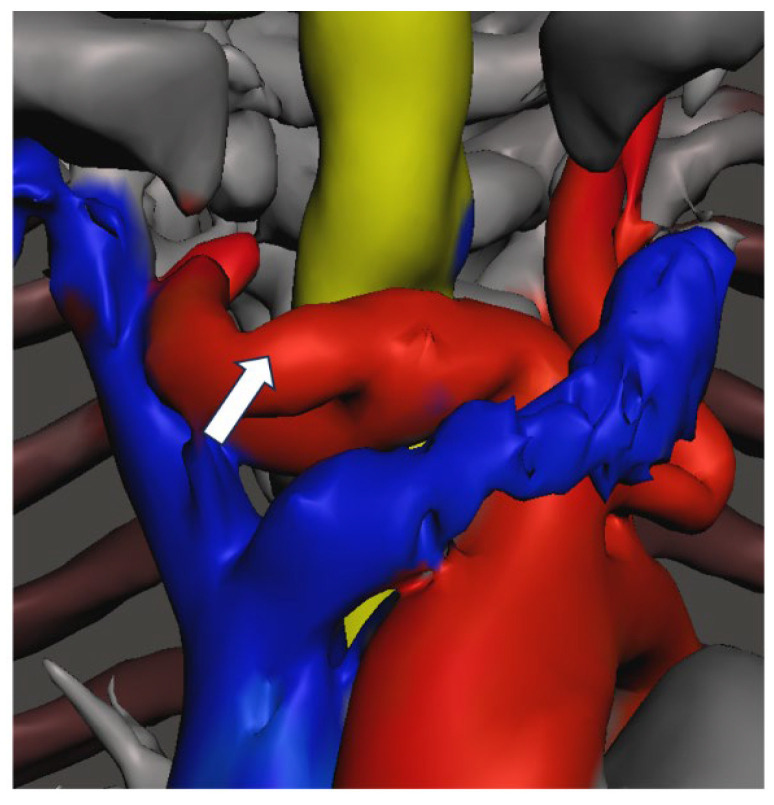
3-dimensional reconstruction of the root of the neck, thoracic inlet, showing an extra loop of truncus brachiocephalus in the upper mediastinum marked with an arrow.

CT imaging of the neck and chest with 3D reconstruction demonstrated a short neck, large head, small thoracic cage, and broad spatulated ribs with reduced intercostal spaces (Figure 1 and Figure 2). The upper airway was bulky with a high anterior larynx, large epiglottis, tortuous trachea and multiple tracheal narrowing (Figure 3). The narrowest tracheal segment measured 3.3 mm in diameter (approximately 50% of the lumen). Patchy bilateral parenchymal opacities were present (Figure 4). An additional loop of the truncus brachiocephalicus arose from the aortic arch and coursed anterior to the trachea at the thoracic inlet, without clear evidence of airway compression (Figure 5).

There was no sputum production, precluding microbiological sampling. A working diagnosis of lower respiratory tract infection due to viral or atypical bacteria was made. Initial supportive management included face-mask oxygen, nebulised adrenaline, corticosteroids, bronchodilators, and broad-spectrum antibiotics. Continuous positive airway pressure was not tolerated due to discomfort with facial interfaces. High-flow nasal oxygen, which bypasses the oral cavity, was better tolerated and marginally reduced oxygen requirements. Despite treatment, his clinical condition gradually worsened, with increasing oxygen requirements and signs of respiratory fatigue. Escalation to intubation and mechanical ventilation was considered. Airway management planning followed the Difficult Airway Society guidelines [13]; incorporating Plan A (endotracheal intubation), Plan B (laryngeal mask airway), Plan C (bag and mask ventilation), and Plan D (front of neck access). Anticipated challenges, potential consequences, and mitigation strategies for each step are summarised in Table 2.

Mechanical ventilation will be challenging due to ongoing infection, broad spatulate ribs with reduced intercostal spaces, and a contracted chest wall with markedly reduced lung volumes, as reflected by the low FEV1 and FVC. Various intubation strategies outlined in Table 2 were considered, including fibreoptic and video-laryngoscopic approaches, and input was sought from clinicians with specialist expertise in MPS. The risks associated with each intervention were discussed thoroughly within a multidisciplinary team and shared with the family. The final option would be palliation, as the risks outweigh the benefits. This would mean that the child will be kept comfortable and all supportive care will be offered, but no active invasive interventions will be done. This would also involve no actively performing cardiopulmonary resuscitation should there be a deterioration. With these options presented, the family was invited for a detailed discussion, during which all risks were explained. Specialist nurses were involved to ensure the family felt fully supported. After careful consideration, the family declined any invasive interventions despite the child’s worsening respiratory status. A collective multidisciplinary decision was therefore made to prioritise comfort and provide palliative care. High-flow nasal oxygen was better tolerated than face-mask oxygen and resulted in a modest reduction in oxygen requirements. Despite comprehensive supportive care, the child sadly passed away peacefully in his sleep after three months of hospital admission.

## 4. Discussion

Maroteaux–Lamy syndrome (MPS VI) is a rare lysosomal storage disorder characterised by arylsulfatase deficiency. This leads to dermatan sulphate accumulation in multiple tissues with significant clinical heterogeneity. Limited awareness among healthcare professionals contributes to delays in recognition and challenges in management. This is a progressive multisystem disease [14], making management complex. The clinical features are short stature, motor dysfunction [15], ocular, oro-dental problems, organomegaly, cardio-respiratory insufficiency, coarse features, umbilical or inguinal hernias, sleep disorder, spinal cord compression, and carpal tunnel syndrome [9]. The ENT problems include adeno tonsillar hypertrophy, hearing loss, otitis media, chronic sinusitis and airway problems [9,11]. The radiological features include dysostosis multiplex, cervical instability, compressive myelopathy, central vertebral beaking, thoracolumbar junctional kyphosis, cervicothoracic kyphosis [15]. These radiological abnormalities further complicate clinical care. Garcia et al. [16] investigated the role of very early and continuous ERT. They have shown that ERT can slow disease progression, preserve endurance and motor function. The authors have also noted a reduction in growth impairment when initiated early. However, ERT does not prevent long-term progression of skeletal or ocular disease. With respect to cardiac disease, the study has shown that cardiac valve involvement generally remained mild, although progression occurred in one patient. These limitations highlight the need for additional therapeutic strategies and continued research into disease-modifying treatments. Airway management remains one of the most challenging aspects of MPS VI care. Anatomical distortion, tracheal narrowing, vascular anomalies, and multisystem comorbidities significantly increase the risks associated with anaesthesia, intubation, extubation, and ventilatory support. Problems with anaesthesia have been noted in case reports as extremely challenging [17]. Although international recommendations on MPS VI published in 2019 [14] provide perioperative guidance, there are no clear protocols for managing acute airway crises. Expert anaesthetic involvement, support from ENT, cardiology, and radiology, and strict avoidance of neck hyperextension have been recommended to minimise the risk of neurological injury. Tracheostomy may be needed when intubation and ventilation are not possible. The other indications of tracheostomy in MPS VI include severe upper airway obstruction, severe sleep apnoea not treatable by CPAP (continuous positive airway pressure) or adenotonsillectomy [14]. Common causes, such as in non-MPS patients needing prolonged ventilation and failure to extubate, are also indications for tracheostomy. Tracheostomy in patients with MPS is recognised as a particularly high-risk intervention due to the combination of a large head, short neck, limited neck extension, and distorted airway anatomy. Selecting an appropriately sized tracheostomy tube can be difficult, and the procedure may be hazardous in the presence of abnormal vascular structures, as demonstrated in our patient, where the risk of catastrophic haemorrhage is significant. Published literature consistently highlights the anatomical challenges associated with tracheostomy in MPS, including an anteriorly displaced larynx, tracheal narrowing and tortuosity, abnormal tracheal cartilage, and difficulty identifying the trachea, all of which increase the likelihood of creating a false passage or failing to secure the airway. Post-procedural complications are also common and include tube obstruction, rapid granulation tissue formation, recurrent inflammation, tracheal stenosis, tube displacement, increased caregiver burden, reduced quality of life, and higher mortality [18,19,20,21]. These factors collectively emphasise why tracheostomy should be approached with extreme caution in MPS and why, in selected cases, it may not be in the patient’s best interests. Following successful intubation, extubation may also be equally challenging and is better performed in a staged fashion. The risks of any intervention need to be evaluated through a multidisciplinary approach that draws on local expertise and, when necessary, input from centres with greater experience. Given the high risk of respiratory deterioration, particularly during infections, personalised management plans and clearly defined ceilings of care with involvement of the family are crucial. Preventive strategies, including infection control measures and immunisation against respiratory infections, may reduce the frequency of acute episodes. Regular monitoring and anticipatory planning support safer elective and emergency interventions. In the end stage of the disease, advanced care planning and palliative approaches ensure that management aligns with the patient’s best interests.

This report has several limitations. Respiratory viral swabs were not performed, which may have helped clarify the underlying infectious trigger. Longitudinal monitoring of urinary GAG levels was also not possible due to a lack of local availability; however, the child demonstrated clear clinical evidence of progressive deterioration, including reduced exercise tolerance, declining mobility, and recurrent respiratory infections. Genetic counselling and family mutation analysis would have been valuable, but could not be undertaken for similar resource-related reasons. These constraints highlight the broader issue of health inequalities frequently encountered in the management of rare diseases [22]. Sleep studies and polysomnography could not be completed because of limited equipment availability and poor patient tolerance. This challenge is well recognised in the literature: polysomnography in MPS is technically difficult to perform [23], often incomplete [18]. Although accurate when successfully obtained, its feasibility in real-world settings is limited [19].

## 5. Conclusions

Maroteaux–Lamy syndrome (MPS VI) is a rare, progressive disorder with multisystem involvement, and respiratory infections can trigger rapid clinical deterioration. Such episodes may result in significant airway compromise and pose substantial risks to health. Careful airway evaluation and coordinated multidisciplinary management are essential when responding to acute crises. Key decisions often involve whether to proceed with interventions such as anaesthesia, intubation, ventilation, extubation, or tracheostomy, each of which must be considered in the context of the patient’s comorbidities. Any intervention should be planned collaboratively by a multidisciplinary team, taking a holistic view of all organ systems and weighing potential risks and benefits. Families should be supported and actively involved in shared decision-making. Personalised care plans should incorporate preventive strategies and outline approaches for both elective and emergency scenarios. Establishing advance care directives and defining ceilings of care are important components of anticipatory planning. In advanced disease, or when the risks of intervention outweigh potential benefits, palliative care should be considered to ensure that management remains aligned with the patient’s best interests.

## Figures and Tables

**Table 1:** Various types of MPS; reproduced from Braunlin et al. [3], who compiled data from Neufeld et al. [4] and Valayannopoulos et al. [5].

MPS Type (Eponym)	Incidence per 10^5^ Live Births; Inheritance Pattern	Typical Age at Diagnosis	Typical Life Expectancy If Untreated	Enzyme Deficiency	GAG
MPS I Hurler (H) MPS I Hurler-Scheie (H-S) MPS I Scheie (S)	0.11–1.67; AR	H: <1 year H-S: 3–8 years S: 10–20 years	H: death in childhood H-S: death in teens or early adulthood S: normal to slightly reduced lifespan	α-L-iduronidase	DS, HS
MPS II (Hunter)	0.1–1.07; XR	1–2 years when rapidly progressing	rapidly progressing: death <15 years, slowly progressing: death in adulthood	iduronate-2-sulfatase	DS, HS
MPS III (Sanfilippo) A-B-C-D	0.39–1.89; AR	4–6 years	death in puberty or early adulthood	heparan sulfamidase (A) N-acetyl-α-D-glucosaminidase (B) acetyl-CoA-α-glucosaminidase N-acetyltransferase (C) N-acetylglucosamine-6-sulfatase (D)	HS
MPS IV (Morquio) A-B	0.15–0.47; AR	1–3 years	death in childhood- middle age	N-acetylgalactosamine-6-sulfatase (A) β-galactosidase (B)	CS, KS (A) KS (B)
MPS VI (Maroteaux-Lamy)	0–0.38; AR	rapidly progressing: 1–9 years, slowly progressing: >5 years	rapidly progressing: death in 2nd–3rd decade slowly progressing: death in 4–5th decade	N-acetylgalactosamine-4-sulfatase	DS
MPS VII (Sly)	0–0.29; AR	neonatal to adulthood	death in infancy- 4th decade **	β-D-glucuronidase	CS, DS, HS
MPS IX (Natowicz) *	unknown	adolescence	unknown	hyaluronidase	CS
MPS X ***	unknown AR	childhood-onset	unknown	Arylsulfatase K	DS

AR: autosomal recessive; CS: chondroitin sulfate; DS: dermatan sulfate; GAG: glycosaminoglycan; H: Hurler syndrome; HS: heparan sulfate; H-S: Hurler-Scheie syndrome; KS: keratan sulfate; S: Scheie syndrome; XR: X-linked recessive. * only 1 patient reported in the literature (Natowicz et al., 1996 [6]); ** death can occur in utero with hydrops fetalis, *** 10 patients reported in the literature (Al Fahdi I et al., 2025 [7]).

**Table 2:** Airway plan as per the Difficult Airway Society guidelines [13] of plan A, B, C and D; the challenges, consequences and methods to mitigate these challenges.

Airway Plan	Challenges	Consequence	Methods to Mitigate
Plan A Endo tracheal intubation	Reduced mouth opening, large tongue, high anterior larynx, and limited neck extension. A tortuous trachea with multiple narrowings can make passage of the endotracheal tube difficult.	Access to the larynx and passage of the endotracheal tube into the trachea will be difficult	Using a small endotracheal tube 1. Nasal intubation 2. Oral intubation with video laryngoscope or Hopkins rod telescope 3. Awake nasal or oral fiberoptic using an airway conduit
Plan B Laryngeal Mask airway (LMA)	Limited mouth opening, large tongue, high anterior larynx, bulky supraglottic	Inserting the LMA and securing a seal will be difficult	Using a reinforced LMA, which is more flexible to reach the anterior larynx
Plan C Bag and mask ventilation	Limited mouth opening, large tongue that can fall posteriorly occluding the airway, bulky oropharynx, bulky supraglottis	Inability to pass oxygen beyond the oropharynx due to obstruction	1. Guedel’s airway to bypass the tongue base 2. Nasopharyngeal airway to bypass the epiglottis
Plan D Front of neck access	Short neck, limited extension, large head, small torso Large vessels in the thoracic inlet	Accessing the cervical trachea will be difficult, a large vessel catastrophic haemorrhage, and inserting the right-sized tracheostomy tube	Avoid tracheostomy; if attempted, perform high tracheostomy. Planning the right tracheostomy tube before the surgery

## Data Availability

All the relevant clinical information regarding this patient has been presented; any additional information may be provided upon request.

## Abbreviations

The following abbreviations are used in this manuscript:

MPS	Mucopolysaccharidosis
ARSB	Arylsulfatase B
HGMD	Human Gene Mutation Database
ENT	Ear, Nose and Throat
GAG	Glycosaminoglycans
ERT	Enzyme Replacement Therapy
BMI	Body Mass Index
FVC	Forced Vital Capacity
FEV1	Forced Expiratory Volume in 1 s
CT	Computerised Tomography
3D	Three Dimensions
LMA	Laryngeal Mask Airway
CPAP	Continuous Positive Airway Pressure
